# Double Zona Drilling for Trophectoderm Biopsy: A Safe Strategy When Inner Cell Mass Herniates

**DOI:** 10.3390/diagnostics16060915

**Published:** 2026-03-19

**Authors:** En-Hui Cheng, Yi-Pin Lin, Maw-Sheng Lee, Chi-Ying Lee, Pin-Yao Lin, Chun-Chia Huang, Wei-Che Lo, Chung-I Chen, Ming-Jer Chen, Chun-I Lee

**Affiliations:** 1 Genetic Diagnosis Laboratory, Lee Women’s Hospital, Taichung 40652, Taiwan; enhuicheng@ivftaiwan.com (E.-H.C.); yipinglin123@ivftaiwan.com (Y.-P.L.); chiyinglee@ivftaiwan.com (C.-Y.L.); 2 Division of Infertility, Lee Women’s Hospital, Taichung 40402, Taiwan; msleephd@gmail.com (M.-S.L.); jellylin0607@gmail.com (P.-Y.L.); agarhuang@gmail.com (C.-C.H.); gjdhjason@gmail.com (W.-C.L.); cci1959@yahoo.com.tw (C.-I.C.); mingjer_chen@yahoo.com.tw (M.-J.C.); 3 Institute of Medicine, Chung Shan Medical University, Taichung 40201, Taiwan; 4 Department of Obstetrics and Gynecology, Chung Shan Medical University Hospital, Taichung 40201, Taiwan; 5 Institute of Bioinformatics and Structural Biology, National Tsing Hua University, Hsinchu 30013, Taiwan; 6 Department of Obstetrics and Gynecology and Women’s Health, Taichung Veterans General Hospital, Taichung 40705, Taiwan; 7 School of Medicine, National Yang Ming Chiao Tung University, Taipei 11221, Taiwan; 8 Department of Obstetrics and Gynecology, School of Medicine, Chung Shan Medical University, Taichung 40201, Taiwan

**Keywords:** blastocyst biopsy, preimplantation genetic testing for aneuploidy (PGT-A), double laser zona drilling, live birth rate, implantation rate

## Abstract

**Background/Objectives**: Laser-assisted zona pellucida (ZP) drilling on day 4 embryos is routinely performed in IVF laboratories to facilitate trophectoderm (TE) herniation for blastocyst biopsy. Nevertheless, inner cell mass (ICM) herniation through the initial ZP opening occasionally occurs and may interfere with standard TE biopsy. **Methods**: This retrospective study assessed the clinical and obstetric safety of a double ZP drilling strategy for TE biopsy in preimplantation genetic testing for aneuploidy (PGT-A) cycles. A total of 560 single euploid embryo transfer cycles were analyzed. Blastocysts were categorized (Groups 1–6) based on ICM/TE herniation patterns and the corresponding biopsy approach. Clinical outcomes were compared between cycles undergoing TE biopsy through a single ZP opening (TE hatching with ICM remaining within the ZP) and cycles requiring a second opening to relocate the biopsy site when the ICM herniated through the original opening or was positioned externally. **Results**: The single-opening approach of Group 1 accounted for 295 cycles (52.7%), with implantation, miscarriage, and live birth rates of 65.4%, 14.0%, and 56.3%, respectively. The double-opening approach of Group 3 was applied in 21 cycles (3.8%), yielding implantation, miscarriage, and live birth rates of 66.7%, 0%, and 66.7%, respectively. No significant differences were observed between the two strategies in implantation, miscarriage, or live birth rates. Obstetric and neonatal outcomes, including gestational age, birth weight, and monozygotic twinning incidence, were comparable. Fifteen healthy infants were delivered following TE biopsy using the double-opening strategy. **Conclusions**: These data support incorporating ICM position into TE biopsy decision-making and suggest that creating a second ZP opening to reposition the biopsy site is clinically feasible and does not compromise reproductive or obstetric outcomes in PGT-A cycles.

## 1. Introduction

Preimplantation genetic testing (PGT) is one of the important techniques in in vitro fertilization (IVF) treatment, and it helps prevent the transmission of genetic diseases. PGT can identify embryos with euploid chromosomes for transfer by screening aneuploidy (PGT-A). Embryonic aneuploidy is prevalent in IVF cycles and leads to implantation failures, early pregnancy loss, and abnormal fetuses. According to the previous evidence, blastocyst biopsy combined with comprehensive chromosomal screening was an efficient framework to increase pregnancy rates per transfer [1,2]. He et al. reported that blastocyst biopsy may not increase additional risk to neonatal outcomes when compared to frozen embryo transfer alone [3].

PGT-A is commonly conducted using trophectoderm (TE) biopsy and vitrified-warmed euploid embryo transfer (ET) in many IVF centers [1,4]. It also enhances the application of single-embryo transfer to reduce multiple pregnancy [5]. The quality of blastocyst with good TE and inner cell mass (ICM) is essential for implantation and development, which is related to a successful pregnancy [6,7]. Previous studies on the effects of embryo grade and cell number of TE biopsy revealed that implantation potential was affected by a lower grade of TE and a larger number of cells retrieved for analysis [8,9]. The biopsy technique to efficiently and securely perform TE sampling of an appropriate cell number, usually 5 to 10 cells, and to avoid damage of ICM is important for PGT.

Currently, there are two main methods of blastocyst biopsy that diverge by the time of zona pellucida (ZP) opening prior to biopsy [10,11,12]. The first method requires the creation of a hole in the ZP by laser on day 3 or 4 embryos. It facilitates TE in herniating through the hole for biopsy on day 5 or 6 [10,13]. The second method is to make a hole in the ZP immediately before biopsy on day 5 or 6 expanded blastocysts [11,14,15]. There is no consensus for which one is superior [16,17]. Both methods have different advantages and disadvantages. Each IVF laboratory may have its preferred method and adjust it along with experience and workflow.

The first method, creating a hole in the ZP on day 3 or 4 embryos, is still commonly used in IVF centers [17]. The major disadvantage is that an embryologist is not able to manipulate whether the hatching portion is TE or ICM on day 5 or 6. Consequently, herniation of ICM instead of TE may happen. In this situation, a second ZP opening for sampling TE away from the ICM provides an alternative strategy to circumvent embryo damage. However, limited data exist on the clinical and obstetric safety of a second ZP opening when ICM herniation compromises the biopsy site. This gap has left embryologists with little evidence-based guidance in such scenarios. There is only a very limited study examining the obstetrical outcome of pregnancy [18]. Safety data regarding the creation of a second hole in the ZP for TE biopsy are mandatory. Therefore, we explore the clinical outcomes of implantation, pregnancy, and live births between groups of one hole and two holes in the ZP resulting from either the original or coping biopsy strategies.

## 2. Materials and Methods

### 2.1. Study Design and Cohort

This retrospective cohort study included IVF/ICSI cycles with PGT-A performed at Lee’s Women’s Hospital between January 2019 and December 2022. Cycles were excluded if maternal age was >45 or <25 years and if they involved repeat IVF attempts, re-biopsy, PGT-M combined with PGT-A, or incomplete transfer data. This study was approved by the Institutional Review Board of Chung Shan Medical University Hospital (IRB: CS1-23027, approved on 20 July 2023).

### 2.2. Ovarian Stimulation, Insemination, and Culture of Embryos

Ovarian stimulation was performed as described previously [19]. The retrieved oocytes were cultured in Quinn’s Advantage Fertilization Medium (Sage BioPharma, Inc., Trumbull, CT, USA) supplemented with 15% Serum Protein Substitute (SPS; Sage BioPharma) in a triple-gas incubator maintained at 5% CO_2_, 5% O_2_, and 90% N_2_. Following fertilization via conventional IVF or ICSI, embryos were cultured in microdroplets of culture medium supplemented with 15% SPS until day 3. Subsequently, embryos were transferred to a blastocyst medium supplemented with 15% SPS and maintained in a group culture system.

### 2.3. TE Biopsy Strategies and Embryo Cryopreservation

**Figure 1:**
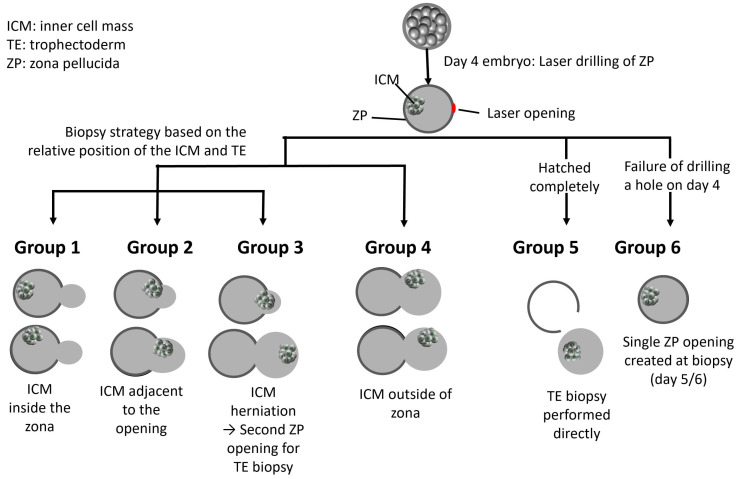
After laser drilling of the zona pellucida (ZP) on day 4, blastocysts were classified into six groups based on herniation patterns of the trophectoderm (TE) and inner cell mass (ICM) on day 5 or 6. Group 1: TE hatching with ICM inside the zona. Group 2: TE hatching with ICM adjacent to the opening. Group 3: ICM herniating through the opening or located above the outside TE. Group 4: ICM outside the zona beside the outside TE. Group 5: Completely hatched blastocysts. Group 6: Failure of zona drilling on day 4. This classification guides the biopsy strategy, with decisions on creating an additional opening based on avoiding ICM injury. The arrow indicates the direction of embryo development from day 4 to the blastocyst stage.

On day 4 post-insemination, a small opening, approximately 10–15 μm, was created in the ZP using laser pulses (OCTAX NaviLase, Vitrolife, Gothenburg, Sweden) to facilitate TE herniation of blastocysts for biopsy on day 5 or 6. TE biopsy was performed when the blastocyst achieved a grade of 4BC or higher, based on Gardner’s scoring system [20]. Blastocysts reaching this developmental stage on day 5 or day 6 were biopsied on the same day according to embryo developmental progression. The procedures were performed in a single session throughout the day. The blastocysts were categorized into six groups according to various herniation portions of TE and ICM (Figure 1): Group 1: TE is hatching, and ICM is inside the zona; Group 2: TE is hatching, and ICM is adjacent to the hole; Group 3: ICM hatches through the hole or at the top of the outside TE; Group 4: ICM is outside the zona at the side of TE; Group 5: ICM is completely hatched; Group 6: failure of zona drilling occurs on day 4. The biopsy strategies regarding the creation of an additional opening or not were decided according to the principle of avoiding ICM injury. If hatching of ICM instead of TE occurred (Group 3), a second zona opening with sampling of TE cells away from the ICM was performed. When Group 6 experienced a drilling failure on day 4, an additional hole was made away from ICM, and TE was biopsied. Groups 1, 2, 4, and 5 can be biopsied directly. The zona drilling failure might be due to the fact that the laser spots did not penetrate the whole zona thickness. A true hole at the zona was not made successfully.

The blastocyst was stabilized using a holding pipette (Humagen, Charlottesville, VA, USA), and 5–10 TE cells were aspirated with a biopsy pipette, utilizing laser pulses of 1.3–1.5 microseconds to facilitate cell detachment. We used the flicking method for biopsy, and this was standardized across groups (Supplementary Video Groups 1–6). Biopsied cells were washed three times in phosphate-buffered saline under a microscope and immediately transferred into RNase/DNase-free PCR tubes for genetic analysis. Following biopsy, blastocysts were cultured in a blastocyst medium supplemented with 15% SPS in a triple-gas incubator for 2 h until vitrification. Vitrification was performed using the Cryotech vitrification method (Repro-Support Medical Research Centre, Tokyo, Japan) according to the protocol described by Gutnisky et al. [21].

### 2.4. Next-Generation Sequencing (NGS) Protocol for PGT-A

Biopsied samples were placed in a lysis buffer, and genomic DNA was fragmented and amplified (SurePlex DNA Amplification System, Illumina, San Diego, CA, USA), following the manufacturer’s guidelines. Whole-genome amplification products of each sample were prepared according to the VeriSeq PGS workflow (Illumina). The purified DNA library was normalized to quantify each sample’s final pooled amount using library normalization additives and purification beads. Normalized samples were pooled, denatured, and sequenced using the Miseq system and reagents (Miseq v3, Illumina). Bioinformatics data were analyzed using BlueFuse Multi Software v4.4 (Illumina), and two technicians re-verified the mosaic chromosomes of each sample. Segmental gains or losses were defined as 10 Mb changes detected by the high-resolution NGS platform [22]. In aneuploid–diploid mosaic ratios, profiles of <20% were considered euploid, and those >80% were considered aneuploid. The whole chromosome mosaic embryos with <50% aneuploid cells, defined as low-level mosaicism, demonstrated significantly better implantation and pregnancy outcomes than those with >50% aneuploid cells of high-level mosaicism [23].

### 2.5. Warming and Frozen Embryo Transfer (FET)

Based on NGS results, aneuploidy blastocysts were excluded from embryo transfer cycles. One euploid blastocyst was selected for each patient in the warming and transfer cycle. If no euploid embryo was available, an embryo with low-level mosaicism would be the second choice. All patients underwent a natural cycle or an artificial cycle using estradiol and progesterone supplementation for endometrial preparation. An endometrial thickness of at least 8 mm must be achieved before FET. Serum β-human chorionic gonadotropin (hCG) was measured 12 days after the transfer. If β-hCG was positive, implantation of a clinical pregnancy was defined according to the presence of a gestational sac on transvaginal ultrasound at 6 weeks of gestation. A miscarriage before 24 weeks was defined as a spontaneous abortion. The live birth was defined as a fetus delivered with heart activity after 24 weeks of gestation.

### 2.6. Statistical Analysis

Categorical variables, including implantation, abortion, live birth, and monozygotic twin rates, were compared using Fisher’s exact test (two-sided) due to the small sample sizes in several groups. Continuous variables were analyzed using one-way ANOVA. Multivariable logistic regression was performed to evaluate live birth outcomes among the six groups after adjusting for maternal age, biopsy day (day 5 vs. day 6), and embryo ploidy status (euploid vs. mosaic). Odds ratios (ORs) and 95% confidence intervals (CIs) were calculated. Pairwise comparisons between groups were assessed using the multivariable logistic regression model. All statistical analyses were performed using SPSS Statistics version 22.0 (IBM Corp., Armonk, NY, USA).

## 3. Results

### 3.1. Cohort Selection and Study Population

**Figure 2:**
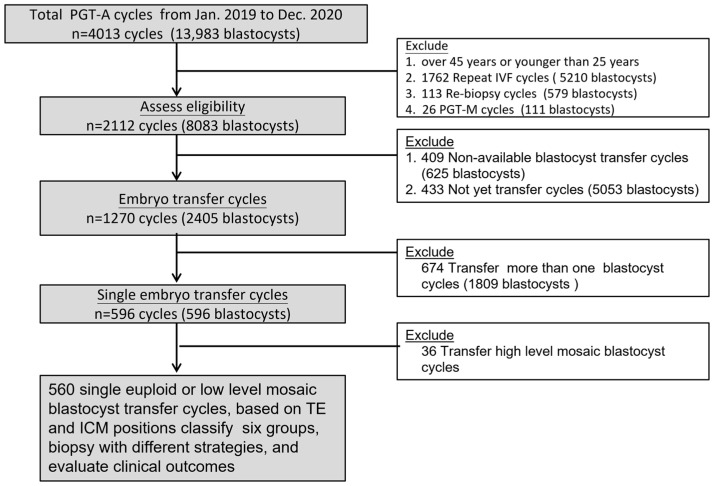
Flowchart of patient selection for analysis of single blastocyst transfer cycles with PGT-A.

A total of 4013 PGT-A cycles (13,983 blastocysts) were initially screened. After applying the eligibility criteria, 1270 transfer cycles involving 2405 blastocysts were included. Cycles with a transfer of ≥2 embryos (*n* = 674) and single-embryo transfers involving high-level mosaic embryos (*n* = 36) were excluded. The final analytical cohort consisted of 560 single euploid/low-level mosaic embryo transfer cycles, including 457 euploid and 103 low-level mosaic blastocysts (Figure 2). The patients were further categorized into six groups according to various herniation portions of trophectoderm (TE) and inner cell mass (ICM), and the blastocysts were biopsied with different strategies (Figure 2). The clinical outcomes were analyzed.

### 3.2. Characteristics of Patients

The characteristics of 560 patients undergoing single-embryo transfer with PGT-A cycles are listed in Table 1. The mean age of women was 36.7 ± 4.4 years, body mass index (BMI) was 22.5 ± 3.9, and duration of infertility was 2.5 ± 2.6 years. The various infertility factors for PGT-A included poor ovarian reserve, advanced female age, recurrent pregnancy loss, repeat IVF failures, male factors, and unexplained infertility, and the number and percentages of patients are presented.

### 3.3. Clinical Outcomes Based on Embryo Herniation Patterns and Biopsy Strategies

The six groups, classified by TE and ICM herniation patterns in day 5 or 6 blastocysts after day 4 ZP laser drilling (Figure 1), had their clinical outcomes summarized in Table 2. There were no significant differences regarding patients’ ages in these six groups. The percentages of biopsy on day 5 were higher than those of day 6 in Groups 1, 2, 3, and 6.

In contrast, the percentages of biopsy on day 6 were higher than those of day 5 in Groups 4 and 5. The distributions of euploid or low-level mosaicism blastocyst transfer were not significantly different among the six groups. In the six groups, Group 1 (TE hatching, ICM inside the zona) had the largest case number (*n* = 295, 52.7%) with a high day 5 biopsy rate (82.0%). The implantation rate was 65.4%, with an abortion rate of 14.0% and a live birth rate of 56.3%. Group 2 (TE hatching, ICM adjacent to the zona opening) had the second largest case number (*n* = 201, 35.9%), with a day 5 biopsy rate of 59.7%. The implantation rate was 59.2%, with an abortion rate of 7.6% and a live birth rate of 54.7%.

**Figure 3:**
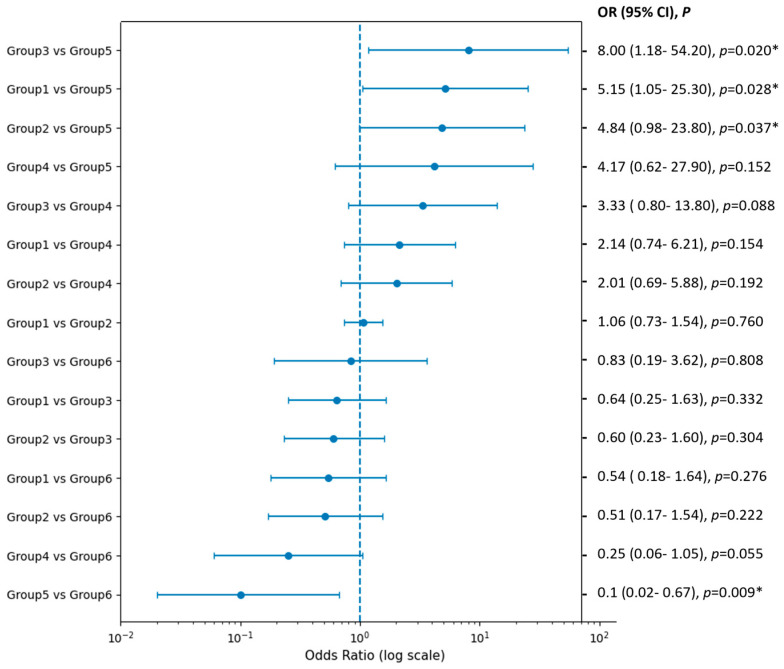
Adjusted odds ratios (ORs) and 95% confidence intervals (CIs) for live birth outcomes among the six blastocyst biopsy strategy groups. Logistic regression analysis was performed with adjustment for maternal age, biopsy day (day 5 vs. day 6), and embryo ploidy status (euploid vs. mosaic). Pairwise comparisons between groups are shown as point estimates (dots) with corresponding 95% CIs (horizontal bars) on a logarithmic scale. The vertical dashed line indicates an OR of 1. Exact OR values, 95% confidence intervals, and *p*-values for each comparison are displayed on the right side of the figure. * indicates statistical significance (*p* < 0.05). *p*-values are presented in italics.

Group 3 (hatching ICM through the zona opening or at the top of the outside TE) had a case number of 21 (3.8%) with a day 5 biopsy rate of 57.1%. The implantation rate was 66.7%, with an abortion rate of 0% and a live birth rate of 66.7%. In order to prevent injury to the ICM, the second hole was created away from the ICM to perform TE biopsy. In univariate analysis, the rates of implantation, abortion, and live birth did not differ significantly among the six groups. Pairwise comparisons using Fisher’s exact test also showed no statistically significant differences among groups. Multivariable logistic regression adjusting for maternal age, day of biopsy, and embryo ploidy status confirmed these findings (Figure 3). Group 1 was used as the reference category in the regression model. The adjusted odds ratios and pairwise comparisons between groups are presented in Figure 3. Group 4 (ICM outside the zona and at the side of outside TE) had a case number of 16 (2.8%) with a day 5 biopsy rate of 37.5%. The implantation rate was 43.8%, with an abortion rate of 14.3% and a live birth rate of 37.5%.

Group 5 (completely hatched out) had a case number of 10 (1.8%) with a day 5 biopsy rate of 10.0%. The implantation rate was 40.0%, with an abortion rate of 50.0% and a live birth rate of 20.0%. The clinical outcomes appeared poorer compared to other groups. The confounding factor of day 6 predominance was noted. The hatched embryo without the protection of ZP may have potential risks associated with biopsy and cryopreservation. These findings raise concern regarding the efficacy of performing biopsies on fully hatched embryos, which may present technical difficulties and increase the risk of compromising embryonic integrity. However, the sample size in this group was too small. The value of TE biopsy for hatched blastocyst may merit further study. Group 6 (zona drilling failure on day 4; re-drill a hole on day 5 or 6) had a case number of 17 (3.0%) with a day 5 biopsy rate of 82.4%. The implantation rate was 70.6%, with an abortion rate of 0% and a live birth rate of 70.6%. These results suggest that delayed drilling remains a viable strategy when earlier access fails. Groups 3 and 6, which involved additional drilling due to either hatching ICM position or initial failure of zona opening, demonstrated comparable implantation and live birth rates. These findings may indicate that flexible biopsy strategies may contribute to good clinical outcomes.

### 3.4. Obstetric Outcomes Based on Embryo Herniation Patterns and Biopsy Strategies

The obstetric outcomes of six groups of single-embryo transfer cycles categorized by various patterns of TE and ICM herniation on day 5 or 6 following zona laser drilling on day 4 are shown in Table 3. The incidences of monozygotic twins, gestational ages, and birth weights were not significantly different between the group with one hole and the group with two holes in the ZP. In Group 3, which has two holes in the ZP, 14 pregnancies were delivered with 15 healthy neonates. Seven cases received non-invasive prenatal tests (NIPTs), one received chorionic villi sampling (CVS), and four received amniocentesis. One-way ANOVA showed no significant differences in gestational ages or birth weights among the six groups. All prenatal tests revealed normal results. The monozygotic twin was delivered at 36 weeks of gestation, with birth weights of 2686 and 1515 g. Mean gestational ages ranged from 37.6 ± 1.7 weeks in Group 3 to 38.3 ± 1.2 weeks in Group 6. Birth weights varied from 2808 ± 421 g in Group 4 to 3467 ± 293 g in Group 5. One-way ANOVA revealed no significant differences in gestational ages or birth weights among the six groups.

## 4. Discussion

For PGT, the blastocyst biopsy method by creating a hole in the ZP using a laser on day 3 or 4 embryos is commonly used in IVF laboratories [10,13]. Compared with cleavage-stage biopsy (day 3), blastocyst-stage trophectoderm biopsy (day 5) allows sampling of multiple cells, providing more DNA for analysis and reducing technical limitations associated with single-cell amplification, such as allele drop-out [24,25]. Moreover, analysis based on a single blastomere may be more susceptible to embryonic mosaicism. Consequently, trophectoderm biopsy has become the preferred approach in contemporary preimplantation genetic testing [25]. However, technical challenges may arise during blastocyst biopsy, particularly when the inner cell mass (ICM) herniates through the zona opening, increasing the risk of inadvertent ICM damage. The double zona drilling strategy proposed in this study may provide an alternative approach to facilitate safe trophectoderm biopsy under such circumstances. Embryos at the morula stage have a wider perivitelline space. We can avoid injuries to embryos from the laser at ZP. The hole of ZP facilitates TE herniation on day 5 or 6, making TE biopsy easier. Nevertheless, the hatching part may be the ICM rather than the TE. The embryologist may need to create an additional hole away from the ICM for biopsy to avoid embryo damage. However, studies regarding the impact of the two holes of ZP for implantation and pregnancy are very limited in the literature. Rubino et al. reported the double zona drilling method, and a patient delivered a healthy baby at 32 weeks of gestation [12]. In our study, analyzing 560 cycles of single-embryo transfer, the rates of implantation, abortion, and live birth between the group with one hole and the group with two holes in the ZP were not significantly different. The incidences of monozygotic twins, gestational ages, and birth weights were also not significantly different. Fifteen healthy babies were delivered in the two-hole group. Our study provides additional clinical evidence regarding the safety data concerning the creation of a second hole in the ZP for TE biopsy.

Montag and Van der Ven investigated the effect of the assisted hatching of embryos in a mouse model. They found that when two holes were created in the ZP either at the zygote or the 2–4 cell stages, the embryos hatched from both openings at the blastocyst stage. Afterward, the embryos were trapped and finally degenerated within the ZP [26]. However, this mouse study of assisted hatching was different from our laboratory procedure of blastocyst biopsy. Initially, we made the first hole in the ZP in a day 4 embryo. And when herniating ICM through the hole occurred on day 5 or 6, the second hole was made for TE biopsy to circumvent injury of the ICM. We observed that all blastocysts collapsed after the biopsy. Then, the biopsied blastocysts were incubated for 2 h before vitrification. We noticed the re-expansion of blastocysts; none of the embryos degenerated, and no hatching occurred in the second hole. The hatching process was seen at the first hole, despite the creation of the second opening. A similar phenomenon had also been found during a 3 h observation after the biopsy carried out by Rubino et al. [18].

The results obtained in this study disclosed that the position of the ICM at the moment of TE biopsy does not affect clinical outcomes. This finding was comparable to the report by Rodriguez-Arnedo et al. [27]. However, in their study, there was a tendency to decrease implantation if ICM was in the herniation position. They thought that, probably due to the manipulation during the biopsy, the ICM would be subjected to more movement and pressure. So, they suggested waiting longer for a biopsy. Instead, we made a second hole for TE biopsy to avoid injury to the ICM. The natural implantation site for human embryos is closer to the ICM, and it may facilitate better implantation chances [28]. However, due to the random drilling of the zona, auxiliary hatching points may be distant from the ICM, potentially reducing the embryo’s implantation probability. Nevertheless, in our study, whether the ICM’s position was closer or farther away from the zona opening, there was no significant difference in implantation and live birth rates.

The size of the zona opening for biopsy is approximately 10–15 μm in our laboratory. We agree that a small opening may result in an increased incidence of monozygotic twins, in view of the significantly higher monozygotic twin rate found in Group 4. In our recent practice, we observed that when the zona opening was created at the time of biopsy on day 5/6 and subsequently enlarged to approximately 30 μm, the embryos appeared to benefit from an assisted hatching effect that may facilitate implantation. However, these observations were not systematically evaluated in the present study and therefore should be interpreted cautiously. Further studies are required to determine whether this technical modification improves clinical outcomes. This approach may represent a potential refinement in biopsy technique that warrants further investigation.

Customarily, we cryopreserve the biopsied blastocyst with a two-hour delay between biopsy and vitrification in our laboratory workflow. We agree to cryopreserve biopsied blastocysts immediately after the biopsy, which avoids re-expansion of blastocysts in view of ESHRE guidelines [24]. Therefore, we have subsequently modified our laboratory workflow to cryopreserve biopsied blastocysts immediately after biopsy in accordance with ESHRE recommendations. This modification was implemented after the study period and was not applied to the embryos included in the present analysis.

Group 4 with the ICM outside the zona exhibited a lower implantation rate, lower live birth rate, and higher abortion rate. Group 4 also had a higher monozygotic twin rate. The time for vitrification in this study was 2 h after biopsy, which may render blastocyst re-expansion difficult for vitrification, especially in Group 4 (with the ICM outside the zona). In addition, an increased possibility of monozygotic twinning may be expected when the ICM is trapped at the height of the TE bridge and when the ZP openings are rather small. Therefore, immediate vitrification after biopsy and standardized ZP openings across groups are needed in this respect.

The frequency of double ZP drilling is rather infrequent. In our series, the incidence was 3.8% (21/560 cases). This is in line with the report of Rubino et al. [18], who reported that the incidence was 3.0% (6/199 cases). Double-zona drilling should be avoided maximally. Two alternatives could be performed: first, by waiting for greater herniation, making the TE more accessible and away from the ICM. The second alternative is to perform simultaneous zona opening and biopsy on day 5. Although both Group 3 and Group 6 involved the creation of an additional zona opening, the underlying reasons were different. Group 3 required a second opening to avoid ICM injury after ICM herniation, whereas Group 6 resulted from technical failure due to the initial day-4 drilling. Therefore, these groups were analyzed separately.

The other method to prevent injury to the ICM is to create a hole in the ZP immediately before the biopsy of day 5 or 6 expanded blastocysts, also mentioned as a non-assisted hatching method [11,15,29]. The advantage is that the procedure can create a hole far from the ICM because the ICM is observable. However, there is no perivitelline space at this stage of the embryo. This method makes it relatively difficult to make a hole without causing injury to the TE of expanded blastocysts. It may sometimes cause blastocyst collapse when making the hole, and it may extend the biopsy time. There is no agreement on which method is better [16,17]. Some authors reported better clinical results with the non-assisting hatching method [12,15]. Zhao et al. reported similar clinical results between the two methods [14]. The recent meta-analysis by Cimadomo et al. revealed that simultaneous zona-pellucida opening and TE biopsy (non-assisted hatching biopsy) allowed better results than the day 3 hatching-based protocol [30]. De Vos and De Munck noted that both approaches are in use, but there is insufficient randomized evidence for preferring one over the other [25]. The superiority of these two methods may deserve further studies to clarify.

### Limitations

This study has several limitations. First, it was a single-center and retrospective study. It may introduce potential selection bias. Second, mosaic embryos were not excluded from the analysis, which may confound the interpretation of implantation and live birth outcomes, as mosaicism itself may influence embryonic developmental potential [23,31]. The third limitation of this study is not only the inequivalent sample sizes across groups but, more specifically, the very low numbers in four groups: Group 3 (hatching ICM at the hole), Group 4 (ICM outside the hole), Group 5 (completely hatched), and Group 6 (failure of zona drilling). These four groups were notably smaller in number than those with the ICM inside the zona (Groups 1 and 2). The relatively small sample size in some subgroups, particularly the double-drilling group (*n* = 21), resulted in wide confidence intervals for several comparisons. Therefore, the absence of statistically significant differences should not be interpreted as evidence of equivalence but rather as reflecting limited statistical precision. The inclusion of low-level mosaic embryos may introduce potential heterogeneity, although their distribution did not differ significantly across groups. This imbalance may have reduced the statistical power to detect subtle differences in clinical outcomes. Future studies with prospective designs with larger and more balanced cohorts are warranted to validate these findings.

While existing evidence generally supports the clinical safety of TE biopsy for PGT, the potential obstetric, neonatal, and long-term child health outcomes following embryo biopsy remain an area of ongoing investigation. A recent scoping review reported that current studies have not demonstrated clear evidence of increased adverse outcomes; however, the available evidence remains limited and heterogeneous, and further long-term follow-up studies are required to better evaluate potential risks [32]. Therefore, continued monitoring of long-term outcomes after embryo biopsy is warranted.

## 5. Conclusions

For PGT, the method for blastocyst biopsy by creating a hole in the ZP on day 3 or 4 embryos is commonly used. Our results underscore the importance of incorporating the ICM position into the decision-making process for TE biopsy on day 5 or 6. When the hatching part is ICM rather than TE, performing a second opening to reposition the biopsy site away from the ICM appears to be a safe and effective strategy. Two laser-drilled openings had no adverse effect on clinical outcomes, indicating procedural flexibility in managing varying hatching conditions. Fifteen healthy babies were delivered via PGT-A, with TE biopsy performed using double laser zona drilling. To our knowledge, this study provides additional clinical evidence regarding the safety of creating a second opening in the zona pellucida for trophectoderm biopsy.

## Figures and Tables

**Table 1:** Characteristics of patients undergoing single-embryo transfer with PGT-A cycles.

Characteristics of Patients	560
Women age (year)	36.7 ± 4.4
BMI	22.5 ± 3.9
Infertility duration (year)	2.5 ± 2.6
**Major Infertility Factors**	
Poor ovarian response	75 (13.4%)
Advanced female age	196 (35.0%)
Recurrent pregnancy loss	59 (10.5%)
Repeat IVF failures	152 (27.2%)
Male factors	18 (3.2%)
Unexplained factors	60 (10.7%)

BMI: Body mass index.

**Table 2:** Clinical outcomes of six groups of day 5 or day 6 blastocysts categorized according to trophectoderm (TE) and inner cell mass (ICM) herniation patterns following zona pellucida (ZP) laser drilling on day 4. Arrows and dashed white circles indicate the regions of interest related to the biopsy procedure.

	Group 1	Group 2	Group 3	Group 4	Group 5	Group 6
	** 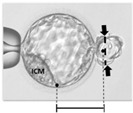 **	** 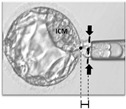 **	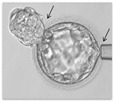	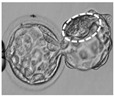	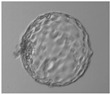	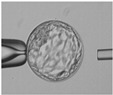
SET cycle, *n* (%)	295 (52.7%)	201 (35.9%)	21 (3.8%)	16 (2.8%)	10 (1.8%)	17 (3.0%)
Age (year)	36.2 ± 4.3	37.3 ± 4.5	37.9 ± 6.3	37.4 ± 3.4	37.5 ± 5.2	36.1 ± 4.0
Percentage of biopsied on day 5	82.0% (242/295)	59.7% (120/201)	57.1% (12/21)	37.5% (6/16)	10.0% (1/10)	82.4% (14/17)
Hole(s) no.	1	1	2	1	1	1
Euploid no.	249	159	14	11	10	14
Low mosaic no.	46	42	7	5	0	3
**Clinical Outcome**						
Implantation rate	65.4% (193/295)	59.2% (119/201)	66.7% (14/21)	43.8% (7/16)	40.0% (4/10)	70.6% (12/17)
Abortion rate	14.0% (27/193)	7.6% (9/119)	0% (0/14)	14.3% (1/7)	50.0% (2/4)	0% (0/12)
Live birth rate	56.3% (166/295)	54.7% (110/201)	66.7% (14/21)	37.5% (6/16)	20.0% (2/10)	70.6% (12/17)

ZP: Zona pellucida; TE: trophectoderm; ICM: inner cell mass; SET: single-embryo transfer; no.: number. Data are presented as a percentage (*n*/*N*) unless otherwise indicated. Group comparisons were performed using Fisher’s exact test (two-sided) due to small sample sizes in several groups. Odds ratios (ORs) and 95% confidence intervals (CIs) were estimated using logistic regression where applicable.

**Table 3:** Obstetric outcomes of six groups categorized by various herniation portions of TE and ICM in day 5 or 6 blastocysts with different biopsy strategies following laser drilling for ZP on day 4.

	Group 1	Group 2	Group 3	Group 4	Group 5	Group 6
SET cycle no.	295	201	21	16	10	17
Monozygotic twin	1.0% (2/193) ^a^	3.4% (4/119)	7.1% (1/14)	14.3% (1/7) ^a^	0% (0/4)	0% (0/12)
Gestational age (week)	38.2 ± 1.9	37.9 ± 2.2	37.6 ± 1.7	38.2 ± 1.8	38.0 ± 0	38.3 ± 1.2
Birth weight (gm)	3079 ± 532	2962 ± 530	2943 ± 379	2808 ± 421	3467 ± 293	2930 ± 462

^a^ indicates *p* < 0.05 by Fisher’s exact test. ZP: Zona pellucida; TE: trophectoderm; ICM: inner cell mass; SET: single-embryo transfer; no.: number.

## Data Availability

The raw data supporting the conclusions of this article will be made available by the authors upon reasonable request, subject to ethical approval and data protection regulations.

## Supplementary Materials

The following supporting information can be downloaded at: https://www.mdpi.com/article/10.3390/diagnostics16060915/s1, Supplementary Video Groups 1–6. Video S1: Representative trophectoderm biopsy procedure for Group 1. Video S2: Representative trophectoderm biopsy procedure for Group 2. Video S3: Representative trophectoderm biopsy procedure for Group 3 with a second zona pellucida opening. Video S4: Representative trophectoderm biopsy procedure for Group 4. Video S5: Representative trophectoderm biopsy procedure for Group 5. Video S6: Representative trophectoderm biopsy procedure for Group 6.
